# Case Report: Dermoscopic, High-Frequency Ultrasound, Contrast-Enhanced Ultrasound Appearances and Special Treatment of a Patient With Syringoid Eccrine Carcinoma on the Chest

**DOI:** 10.3389/fonc.2021.717581

**Published:** 2021-11-15

**Authors:** Jing Zhang, Xun Liu, Mo Zheng, Jing Yin, Weibin Xing

**Affiliations:** ^1^ Department of Dermatology, The Fifth Central Hospital of Tianjin, Tianjin, China; ^2^ Department of Ultrasound, The Fifth Central Hospital of Tianjin, Tianjin, China; ^3^ Department of Pathology, The Fifth Central Hospital of Tianjin, Tianjin, China

**Keywords:** dermoscopy, high-frequency ultrasound, contrast-enhanced ultrasound, diagnosis, treatment, sweat gland neoplasms

## Abstract

This article aims to explain the use of a variety of noninvasive of minimally invasive examinations to obtain reliable diagnostic clues. The choice of treatment methods and repair techniques for wound defects are also critical in terms of the prognosis. Here, we describe the case of a 53-year-old male patient who visited our dermatology clinic due to a red plaque on the inner side of his left nipple without any symptoms for more than 30 years. He was given dermoscopy, high-frequency ultrasound (HFUS), Color Doppler flow imaging (CDFI), and contrast-enhanced ultrasound (CEUS) examinations. Currently, there are no literatures on these auxiliary examinations for this disease. Dermoscopy revealed that there were abundant blood vessels on the periphery of the skin lesion with obvious dilation. HFUS revealed an inhomegeneous hypoechoic solid mass in the dermis with clear borders and irregular shape. CDFI indicated that there are abundant blood flow signals in the periphery and central of the tumor. CEUS showed a mixed inhomogeneous, grid-like high-enhancement pattern. Based on the above auxiliary findings, the possibility of malignant lesion was suspected. Therefore, the patient was given a pathological examination, which showed that many luminal structures of the dermis layer were embedded in the hyperplastic fibrous tissue. The atypical cells were not obvious but showed an infiltrating growth pattern. Immunohistochemistry showed positive reaction for cytokeratin 7 (CK7), epithelial membrane antigen (EMA), and carcinoembryonic antigen (CEA) and a weak positive results was obtained for S-100. There was also a negative result for CK20, gross cystic disease fluid protein 15 (GCDFP-15), and P63. As a result, the patient was diagnosed with “syringoid eccrine carcinoma.” The treatment was surgical excision. Mohs microsurgery was combined with the looped, broad, and deep-buried suturing technique (LBD tension-reduced suturing technique). This technique directly sutures the wound instead of carrying out traditional skin grafting or flap transfer. The postoperative follow-up results were satisfactory as no obvious keloid formed on the wound during the follow-ups. In conclusion, ultrasound is greatly advantageous in tumor morphology and hemodynamics. It orients the therapeutic management and assesses the therapeutic efficacy and the tumoral prognosis. In surgical treatments, a less-traumatic operation should be selected to reduce the patient’s pain.

## Introduction

Syringoid eccrine carcinoma (SEC) is an infiltrative, low-grade malignant tumor derived from the eccrine glands, and it is rarely encountered in the clinic. This study describes a patient with SEC in the chest area from the aspects of pathology, dermoscopy, high-frequency ultrasound (HFUS), contrast-enhanced ultrasound (CEUS), and color Doppler flow imaging (CDFI), as well as its unique treatment. The manifestations of HFUS and CEUS of this disease have never been described in any literature, and we shall be able to learn more through this case study.

## Patient Information

**Figure 1:**
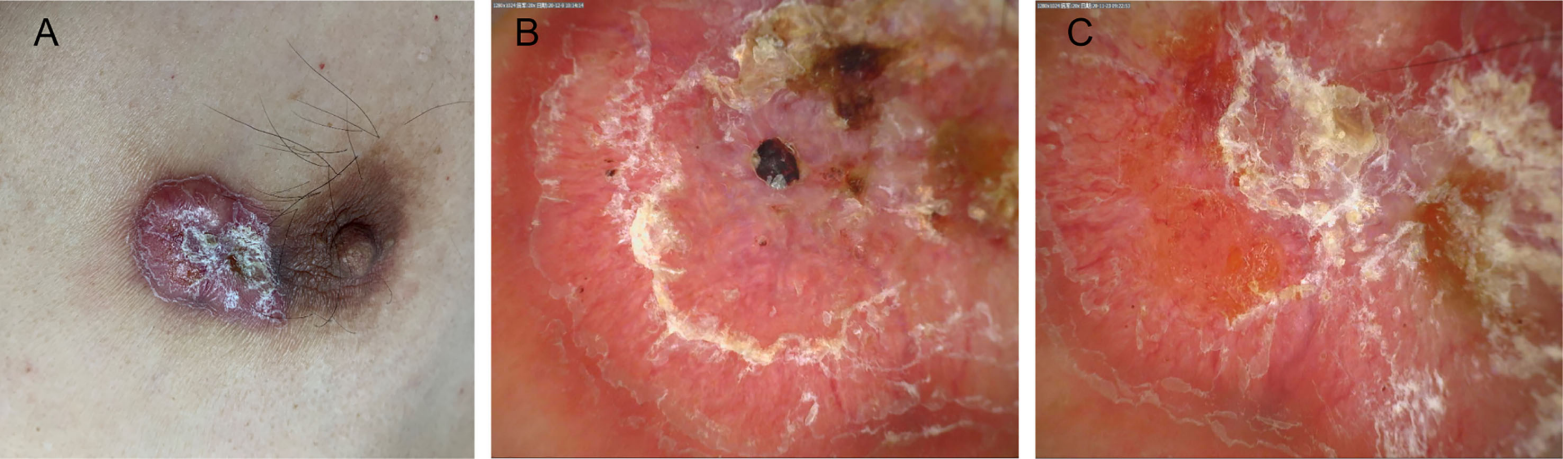
**(A)** An irregular plaque with a scab on the inside of the left nipple. **(B, C)** The dermoscopic findings included a bright red background, apparent central keratosis with erosion and blood scabs, and tortuous dilation of peripheral dendritic vessels at the edge (×20).

The patient was a 53-year-old man. Thirty years ago, the patient had a mung bean-sized red papule on the inner side of his left nipple without obvious inducement. The papule gradually increased in size, but there were no conscious symptoms. In the past year, the patient experienced erosion and a scab appeared in the center of the lesion. As a result, he visited our clinic (**Figure 1A**). There was no family history with similar symptoms. The patient had tuberculosis in the right chest wall 30 years ago and was cured back then.

## Clinical Findings

An irregular, red, annular plaque that had a hard texture was on the patient’s inner side of the left nipple, and it was approximately 2 × 3 cm in size. There were exudations of yellow and blood crusts on the surface of the skin lesion but there were no tenderness and squeezing pain. The physical examination, including palpations of the lymph nodes, chest, and abdomen, was unremarkable.

## Diagnostic Assessment

Routine laboratory examination revealed no abnormality. Ultrasound revealed no abnormal enlargement of the bilateral axillary, supraclavicular, and cervical lymph nodes. No abnormalities were found in the left papilla area. Chest computed tomography illustrated the presence of multiple sites of inflammation in both lungs, as well as right pleural hypertrophy with calcification. Abdominal ultrasound disclosed fatty liver, the rest were normal. Colonoscopy revealed no abnormality.

Regarding the polarization pattern of dermoscopy, mild-to-moderate tortuous dilation of peripheral radial dendritic vessels were visible under a bright red background together with yellowish white scales, erosion, and a blood scab in the center of skin lesion (**Figures 1B, C**).

**Figure 2:**
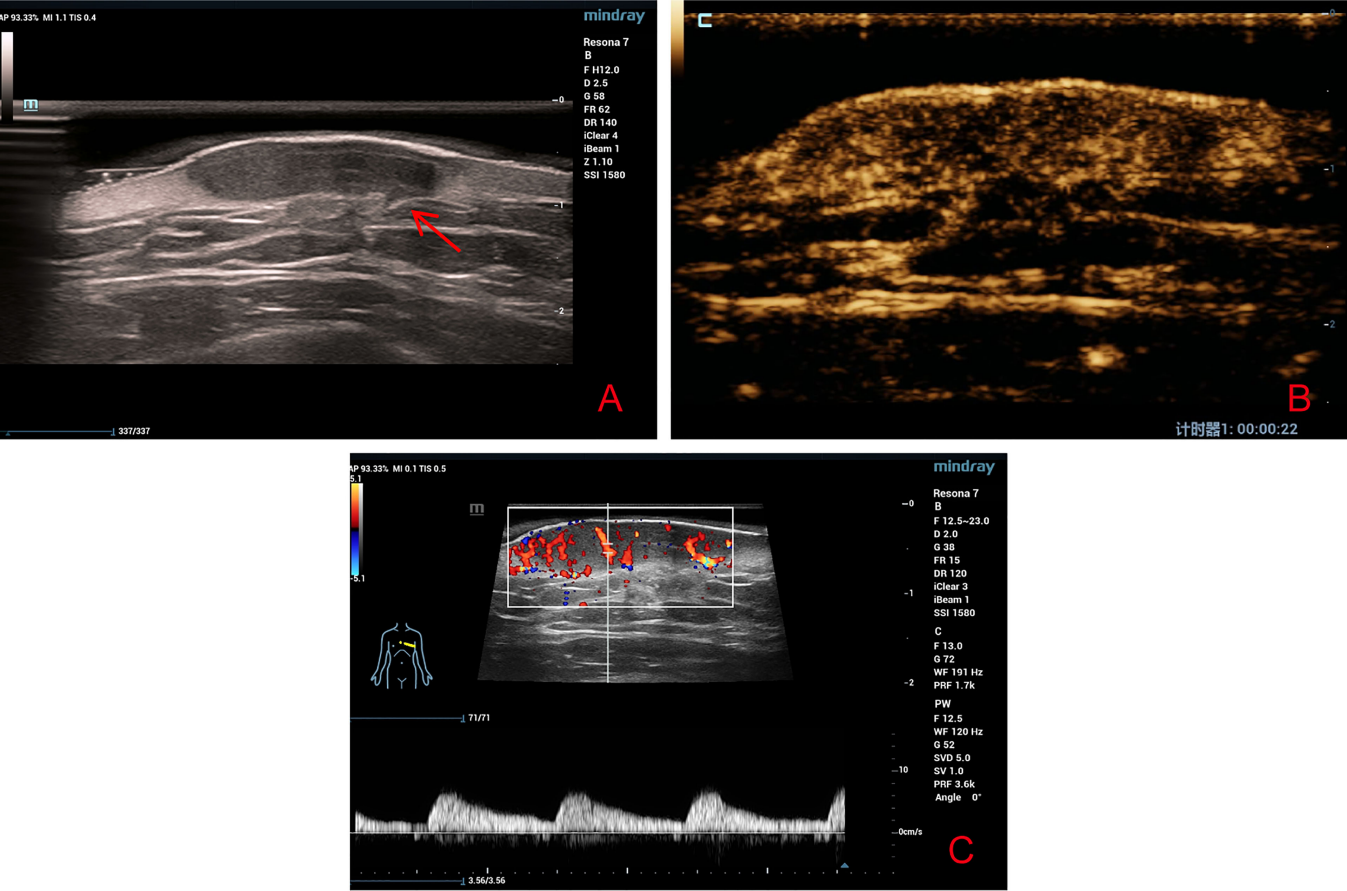
**(A)** High-frequency ultrasound revealed a well-defined irregular hypoechoic solid tumor in the dermis with protruding structures at some area that looked like a beak of a bird (red arrow). **(B)** Contrast-enhanced ultrasound revealed a heterogeneous high-enhancement pattern. **(C)** Color Doppler flow imaging revealed abundant blood flow signals around and inside the tumor.

HFUS revealed an inhomogeneous hypoechoic solid mass in the skin dermis that was approximately 2.8 cm × 1.9 cm × 0.6 cm in size with a clear boundary. It had an irregular shape with protruding structures at some area that looked like a beak of a bird (**Figure 2A**). CDFI illustrated the presence of abundant blood flow signals in the periphery and central of the tumor while the Adler blood flow grade was III. The peak systolic velocity was 6.66 cm/s, the end diastolic velocity was 1.88 cm/s, the resistive index was 0.72, and the spectrum presented high speed and high resistance (**Figure 2B**).

After communicating with the patient, he agreed to undergo CEUS and signed the consent form. In CEUS, the contrast agent (SonoVue, Bracco, Italy) began to enter the lesion within 15 s, and the intensity peaked at 22 s, displaying a mixed inhomogeneous, grid-like high-enhancement pattern. The baseline intensity and peak intensity were 6.60 and 18.38 dB, respectively (**Figure 2C**). Based on CDFI, HFUS, and CEUS findings, the possibility of malignant lesions was suspected.

**Figure 3:**
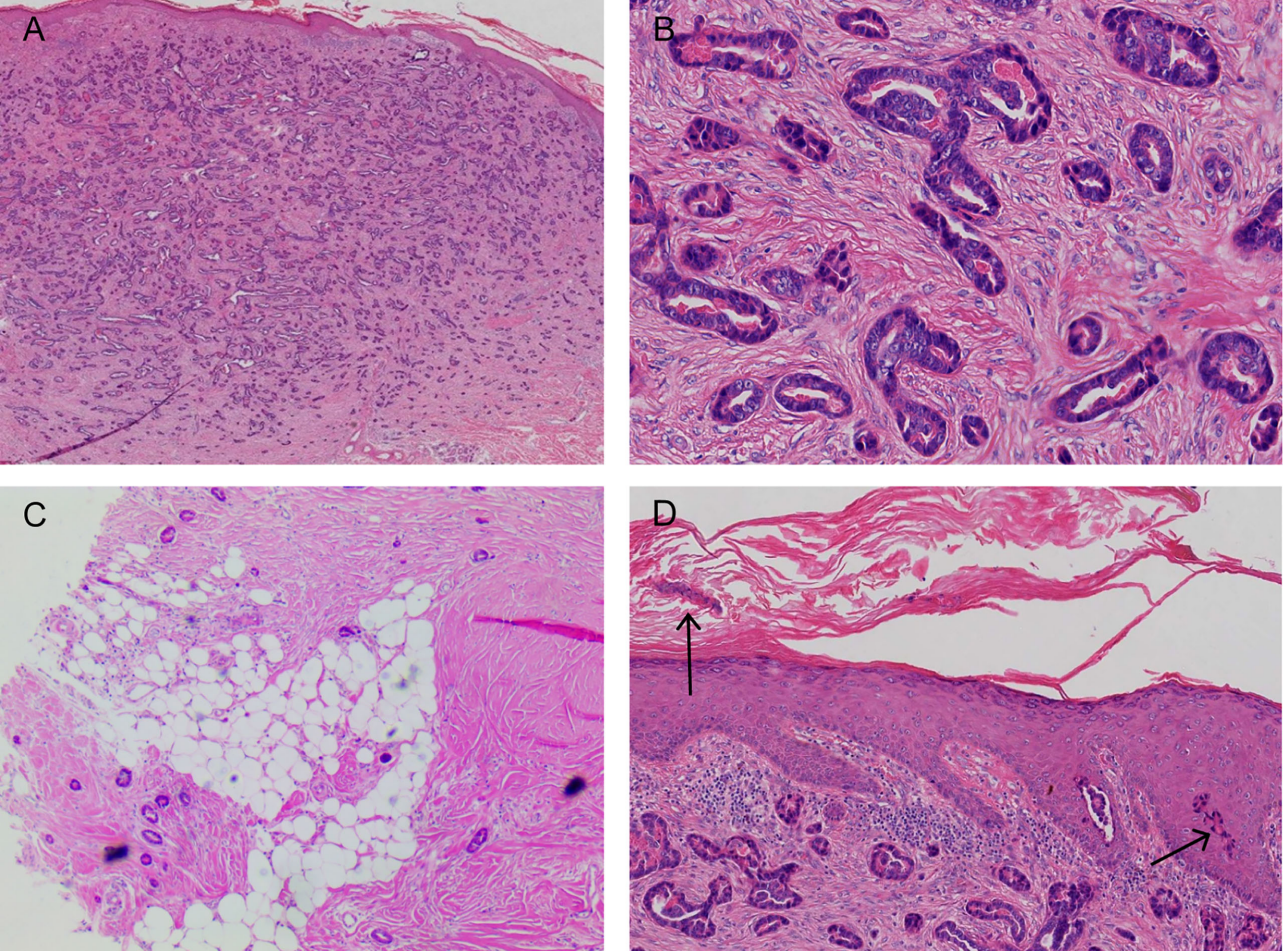
**(A, B)** A large number of densely distributed ductal and cystic lumen-like structures in the whole dermis and mucus-like substances were visible in some lumina. The tumor cells were small, with round nuclei and occasionally karyokinetic images. **(C)** The tumor tissue infiltrated the fat layer (black arrow). **(D)** A few tumor cells nested in the epidermis (black arrow; hematoxylin and eosin: **A**, ×40; **B**, ×200; **C**, ×40; **D**, ×100).

**Figure 4:**
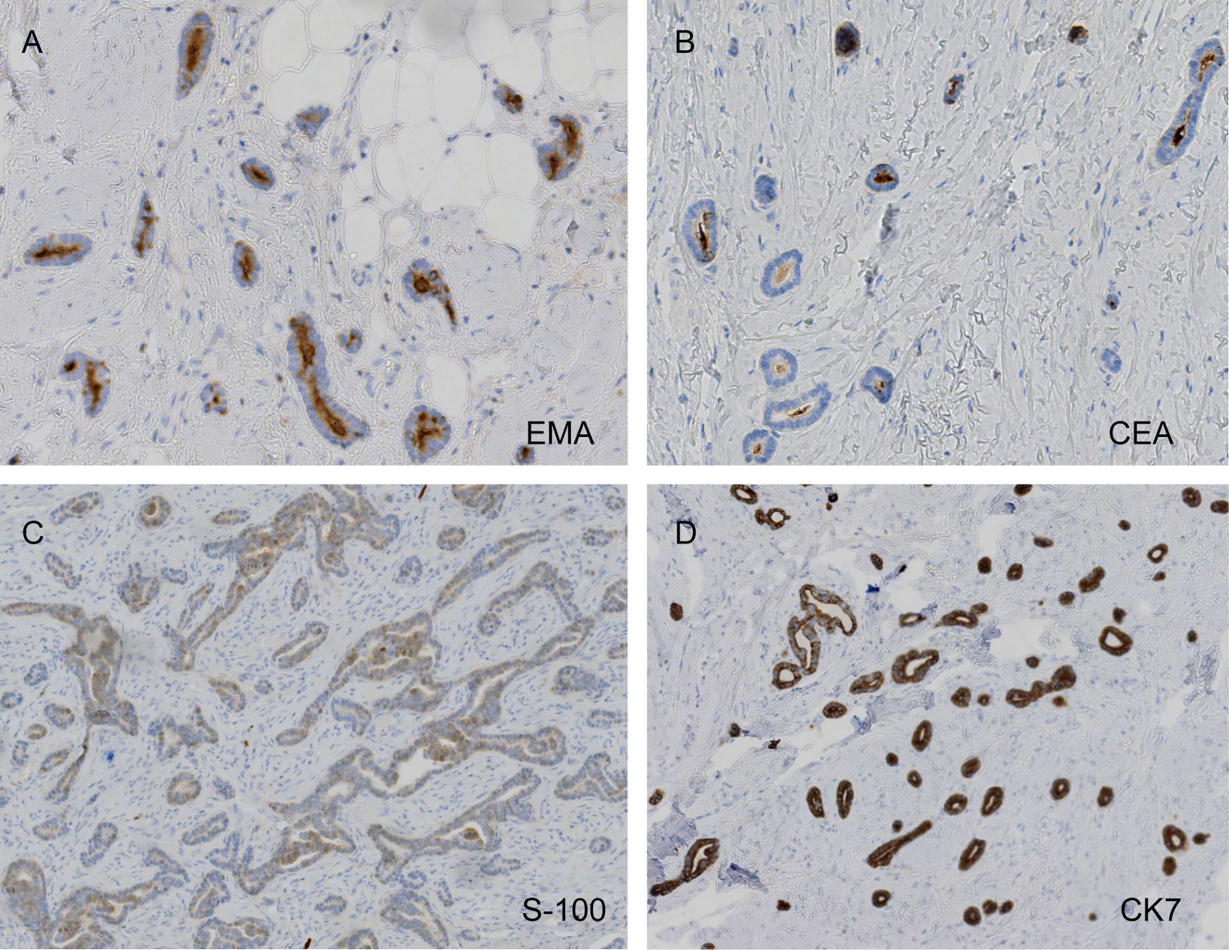
Carcinoembryonic antigen (CEA) and epithelial membrane antigen (EMA) positivity, weak protein S-100 weakly positivity, and diffusely strong cytokeratin 7 (CK7) positivity (**A**, EMA, ×200; **B**, CEA, ×200; **C**, S-100, ×100; **D**, CK7, ×100).

The pathological examination illustrated the presence of full-thickness fibrous tissue hyperplasia in the dermis, in which round and irregular glandular ducts and luminal structures were densely distributed. The glandular epithelial cells were small; the nuclei were round and hyperchromatic while the nucleolus was obvious. The cytoplasm was eosinophilic, occasionally with mitotic images, and an eosinophilic red substance was visible in the glandular cavity. Tumor infiltration was found in both epidermal and subcutaneous tissue. No tumor thrombi were present in the vessels, and no keratinous cysts and cribriform structures were found (**Figure 3**). Immunohistochemistry, cytokeratin 7 (CK7), epithelial membrane antigen (EMA), and carcinoembryonic antigen (CEA) were all positive, S-100 was weakly positive, and the Ki-67 index was approximately 5%–30%. CK20, gross cystic disease fluid protein 15 (GCDFP-15), and p63 were all negative (**Figure 4**). The results of dermoscopy, CDFI, HFUS, CEUS, and pathology were consistent with a diagnosis of SEC.

Before providing a SEC diagnosis, the mass had to be distinguished from sclerosing basal cell carcinoma (BCC), primary cutaneous adenoid cystic carcinoma (PCACC), microcystic adnexal carcinoma (MAC), and skin metastases of visceral adenocarcinoma. The distinction could be done mainly based on pathological manifestations, because their HFUS, CDFI, and CEUS manifestations have not been described in any literatures. The pathological feature of SEC is the tadpole-like and trabecular epithelial components that are embedded in the collagen fibrous matrix; this is similar to syringoma, but the slight atypia and infiltrating growth pattern of the cells can be distinguished between both. SEC lacks horn cyst, hair follicle differentiations, and pleomorphic mesh-like structure, which makes it distinguishable from MAC and PCACC. However, it is sometimes difficult to distinguish sclerosing eccrine carcinoma from PCACC. Positive results were obtained for tumor EMA and CEA, but the cells around the mass were not arranged in a fence-like manner and there were no contraction gap, which makes it distinguishable from BCC. The skin metastasis of visceral adenocarcinoma, combined with clinical manifestations, laboratory examinations, imaging examinations, negative CK20 and GCDFP-15 ruled out this possibility (1–3).

## Therapeutic Intervention

**Figure 5:**
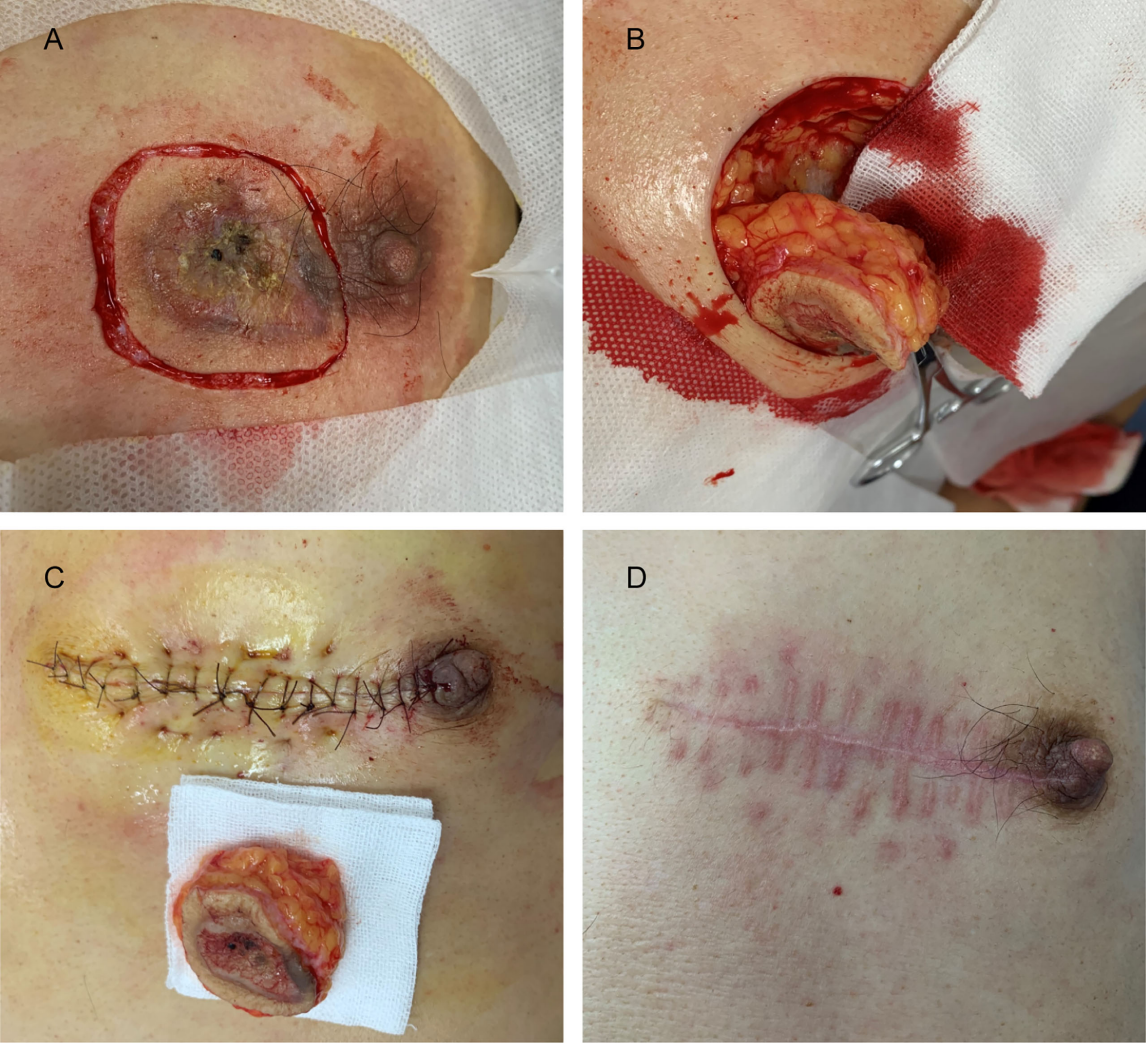
**(A)** The margin of the skin lesion was expanded by 0.5 cm to demark the resection area. **(B)** The resection reached the deep layer of the superficial fascia. **(C)** Image taken immediately after LBD tension-reduced suturing was completed. **(D)** Three months after surgery.

**Figure 6:**
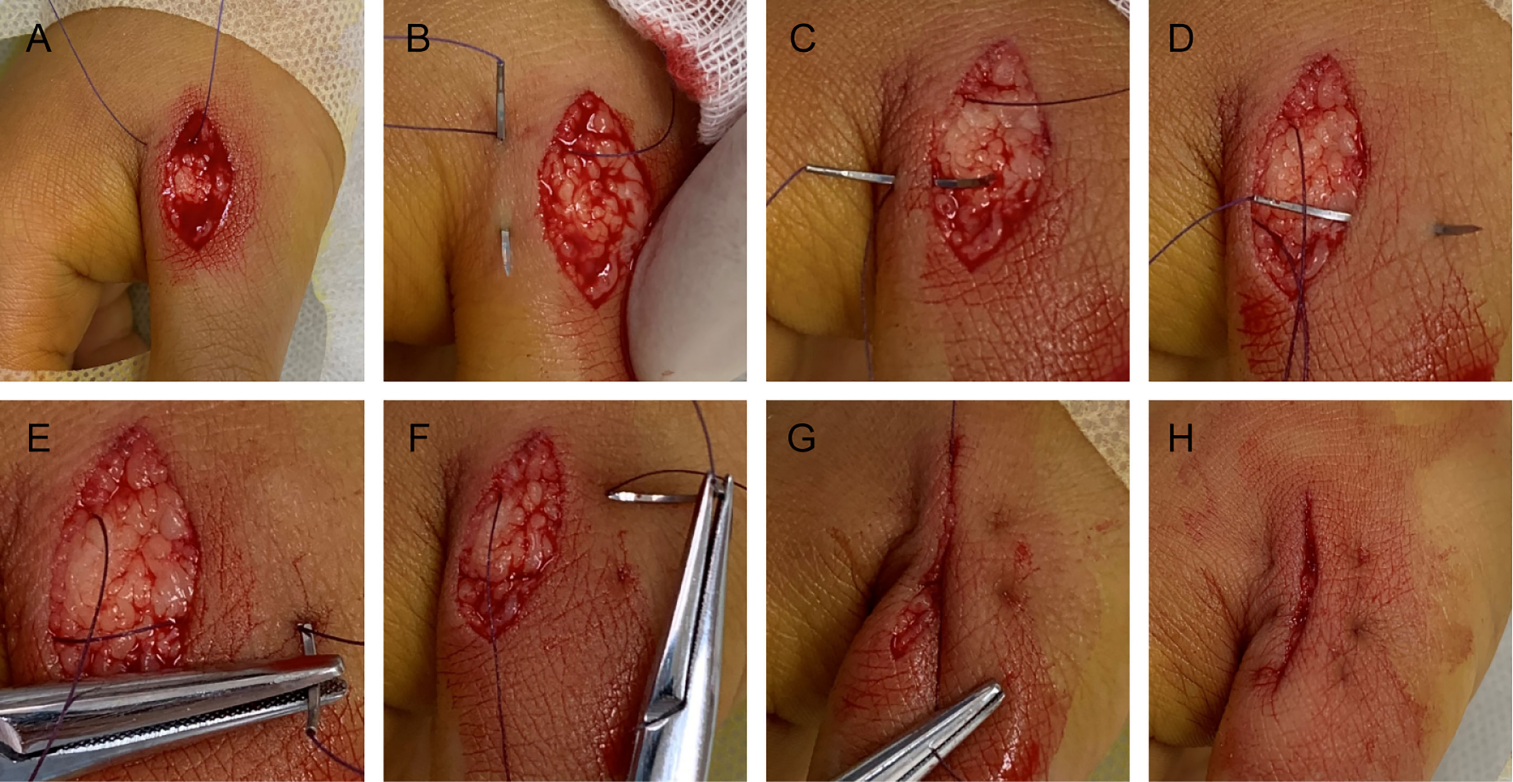
Detailed operation steps of LBD tension-reducing suture follow the panels **A–H**.

The pathological and auxiliary examinations did not indicate the possibility of metastasis, thus, the patient was treated with a wide local excision; Mohs microsurgical resection was planned (**Figure 5**). A 0.5-cm range away from the edge of the skin lesion was marked as the resection area. After fully disinfected with iodophor, the lesion was anesthetized layer by layer. Along the marked range, the skin lesion tissue was deeply excised to reach the deep layer of the superficial fascia before completely removing the mass. The wound was thoroughly inspected and covered with iodophor gauze as an open treatment procedure as the patient awaited the following analysis. After the incisal edge of the skin lesion was marked with 12 points, the pathologist took samples from the incisal edge of the skin, corresponding circumferential soft tissue, and basal margin according to the marked points. The samples were quickly frozen and stained before they were placed under microscopic observation. When all sites showed negative results, the wound was directly sutured based on the markings instead of using skin flap transfer and skin graft repair. The subcutaneous tissue was sutured with absorbable suture, and the LBD tension-reduced suturing technique was used to suture the dermal tissue to ensure that the bilateral skin edges were completely closed and slightly everted (detailed operation steps are shown in **Figure 6**). Finally, the intradermal tissue and epidermal tissue were sutured. After the completion of the surgery, further iodophor disinfection was conducted, and cotton pad bandaging was performed to provide pressure to the wound. In order to prevent infection, dressings were regularly changed (**Figure 5**).

## Follow-Up and Outcomes

**Figure 7:**
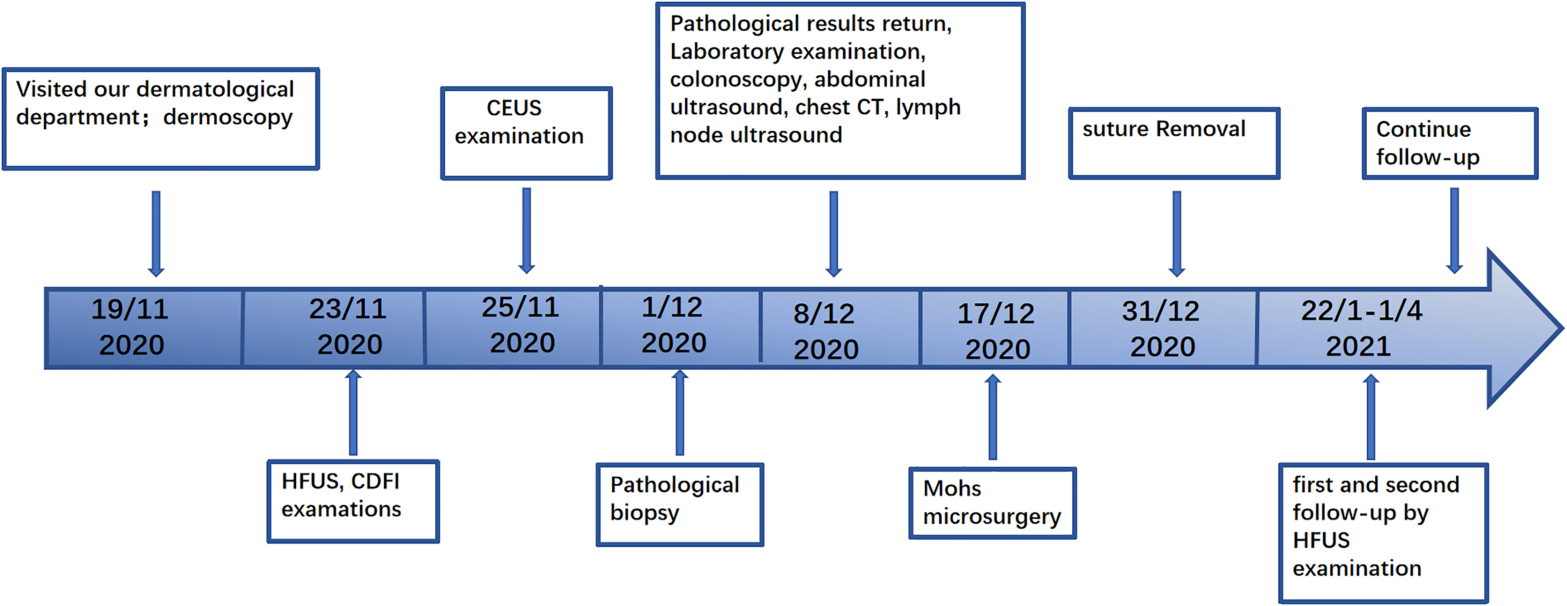
Timeline of the patient diagnosis and treatment course.

The patient experienced a slight pain in the postoperative wound that disappeared after 1 month. The sutures were removed 2 weeks after surgery. Postoperative follow-up was performed during the 1st month and 3rd month, and no space-occupying lesions were found during HFUS examinations. No keloid was found (**Figure 5**). No adverse events occurred during the follow-ups. The patient continued with routine follow-up every 3 months. A chronological summary of the case report is shown in **Figure 7**.

## Discussion

SEC is a rare malignant skin adnexal tumor accounting for less than 0.01% of all malignant skin tumors and lacks a clear pathogenesis (4–6). The typical pathological feature of this disease is that differentiated basal-like cells are embedded in the dense and transparent fibrous matrix, and they invade the subcutaneous fat, fascia, or skeletal muscle. Another common feature of the disease is neural invasion, which may be related to the recurrence tendency of the tumor (7). This case featured no neural invasion, which may have resulted in a better prognosis. Although there were no distinctive appearances for this disease under dermoscopy, the number and the extent of dilatation of blood vessels were noticeable. HFUS illustrated that the local tumor tissue infiltrated the adipose layer. CDFI revealed the presence of abundant blood flow signals, mainly distributed in the center of the tumor. This was significantly different compared with keloids, where the blood flow signals were located mainly at the peripheral area and less in the central area. As a microvascular imaging technique, CEUS can reveal a clearer image of small vessels that are <100 µm in diameter which in turn can show a clearer image of blood vessel distribution compared with CDFI. It allows for observer to observe the perfusion and regression of capillaries which results in a higher accuracy for evaluating benign and malignant tumors (8–11). Crisan conducted US and CEUS to 18 patients with malignant skin tumors and five patients with benign skin tumors. The results illustrated that malignant tumors were hypoechoic on US, whereas they had a diffuse inhomogeneous perfusion pattern on CEUS (12). The peak time and regression time of the contrast agent were faster than those of benign tumors, highlighting the characteristics of “fast in and fast out” (10). The performance of CEUS in this case was consistent with this conclusion. This was possibly because the large amount of neovascularization in the malignant tumor resulted in abnormal blood flow. The characterization of neovascularization includes thin walls, a small lumen, absents of muscle layer, and compression of peripheral blood vessels. Therefore, peripheral blood vessels are distorted and even the normal blood vessel distribution is destructed, resulting in abnormal hemodynamics (13). In general, these auxiliary examinations, especially HFUS combined with CEUS, are of great significance for detecting benign and malignant tumors. It is generally recommended to carry out the US and CDFI first, followed by CEUS if necessary. Certainly, an experienced sonographer is still required if you wish to identify the clues to whether the tumor is benign or malignant with these auxiliary examinations, especially the US and CEUS examinations. As there are not enough relevant literatures reporting on CEUS examination of skin tumors, we still need more cases to research and understand. At the same time, the price of contrast media agent is more expensive, limiting the use of such media for patients. In any case, pathological biopsy is the key for a final diagnosis, which cannot be replaced by any examination. However, the exploration of US and CEUS examinations for rare diseases is still extremely meaningful. In conclusion, this case report attempts to remind dermatologists that we should try to use more noninvasive or minimally invasive auxiliary examinations to help diagnose our patients.

Presently, most patients undergo Mohs microsurgery, and skin flap repair or skin grafting is used to repair the wound defect (14). The patient in this study underwent Mohs surgery, which could avoid unnecessary damage caused by blind excision in expansion and depth. However, the wound defect of this patient was not repaired by conventional skin grafting or flap transfer repair, but it was sutured directly. The LBD tension-reduced suturing technique recommended by Professor Yang Dongyun in China was used to suture the wound (15). The method was modified based on the surgical horizontal mattress tension-reduced suturing technique. The suture passes through the dermis in part to form a rectangular track, and the suture is built in so that the incision tension is reduced according to the use of the absorbable suture. Direct sutures can avoid additional incisions caused by skin grafting or flap repair. The most important finding was that the new tension-reduced suturing technique can minimize scar generation, which could be of a high importance based on the position of the lesion on the anterior chest. Additionally, the effective reduction of tension also shortens the time for removal of the suture, reduces the rest time, and alleviates several inconveniences of the patient.

## Data Availability Statement

The original contributions presented in the study are included in the article/supplementary material. Further inquiries can be directed to the corresponding author.

## Ethics Statement

Written informed consent was obtained from the individual(s) for the publication of any potentially identifiable images or data included in this article.

## Author Contributions

JZ and WX carried out the operation and collected data. JZ drafted the manuscript. XL conducted ultrasound examination and diagnosis. MZ and JY were responsible for pathological section, staining, and diagnosis. All authors contributed to the article and approved the submitted version.

## Conflict of Interest

The authors declare that the research was conducted in the absence of any commercial or financial relationships that could be construed as a potential conflict of interest.

## Publisher’s Note

All claims expressed in this article are solely those of the authors and do not necessarily represent those of their affiliated organizations, or those of the publisher, the editors and the reviewers. Any product that may be evaluated in this article, or claim that may be made by its manufacturer, is not guaranteed or endorsed by the publisher.
